# RNA Demethylase FTO Mediated RNA m^6^A Modification Is Involved in Maintaining Maternal-Fetal Interface in Spontaneous Abortion

**DOI:** 10.3389/fcell.2021.617172

**Published:** 2021-07-19

**Authors:** Weiyu Qiu, Yuexi Zhou, Haiwang Wu, Xiaoli Lv, Lilin Yang, Zhenxing Ren, He Tian, Qingying Yu, Jing Li, Weixian Lin, Ling Zhao, Songping Luo, Jie Gao

**Affiliations:** ^1^Guangzhou University of Chinese Medicine, Guangzhou, China; ^2^Department of Gynecology, The Affiliated Hospital of Guangzhou University of Chinese Medicine, Guangzhou, China; ^3^Shanghai Key Laboratory of Diabetes Mellitus, Center for Translational Medicine, Shanghai Jiao Tong University Affiliated Sixth People’s Hospital, Shanghai, China; ^4^Academy of Integrative Medicine, Shanghai University of Traditional Chinese Medicine, Shanghai, China

**Keywords:** villous, spontaneous abortion, FTO, m^6^A, RNA methylation

## Abstract

The N6-methyladenosine (m^6^A) RNA modification regulates the expression of genes associated with various biological and pathological processes, including spontaneous abortion (SA). The aim of this study was to determine the role of the m^6^A demethylase fat mass and obesity (FTO)- associated protein in SA. The *FTO*,*IGF2BP1* and *IGF2BP2* mRNA levels were significantly lower in the chorionic villi obtained from spontaneously aborted pregnancies compared to that of normal pregnancies, while the expression levels of *METTL3* and *WTAP* were significantly elevated. However, *ALKBH5*, *YTHDF2*, and *IGF2BP3* were elevated with no statistical significance between groups. In addition, MDA was elevated and SOD levels were decreased in the villi tissues of the SA group compared to the normal group, which was indicative of placental oxidative stress in the former. Furthermore, the expression of FTO and HLA-G were significantly decreased in the trophoblasts of the SA patients compared to that of normal pregnant women, while that of m^6^A was markedly higher in the former. In addition, the *HLA-G* and *VEGFR* mRNA levels were downregulated in the SA versus the control group, and that of *MMP2*, *MMP7*, *MMP9* and *VEGFA* were upregulated. Finally, The RIP assay showed significantly decreased levels of FTO-bound *HLA-G*, *VEGFR* and *MMP9* RNA in SA patients (*P* < 0.05), which corresponded to an increase in transcripts enriched with the m^6^A antibody (*P* < 0.05). However, compared with normal pregnant women, the levels of *HLA-G*, *VEGFA*, *VEGFR*, and *MMP2* mRNA bound by YTHDF2 were significantly decreased in SA patients. Compared to the normal pregnant women, both FTO- and m^6^A-bound *MMP7* were significantly increased in SA patients (*P* < 0.05), but YTHDF2 almost unbound to *MMP7* mRNA. In summary, the downregulation of FTO in the chorionic villi disrupts immune tolerance and angiogenesis at the maternal-fetal interface, resulting in aberrant methylation and oxidative stress that eventually leads to SA.

## Introduction

Spontaneous abortion (SA) is the most common complication during the first trimester of pregnancy with an incidence rate of 10–15% (Sahin et al., 2001). The maternal-fetal interface, which is the site of nutrient exchange and circulation between the placenta and the growing fetus, has been implicated in miscarriage and preeclampsia. A dysfunctional maternal-fetal interface induces oxidative stress in the placenta and the subsequent loss of placental synthetic trophoblast cells, which contributes to the pathogenesis of abortion and eclampsia (Jauniaux et al., 2003). Studies increasingly show that normal progression of pregnancy relies on the dynamic balance between oxidases and antioxidant enzymes, and any disruption in this balance leads to pathological complications such as SA (Mentese et al., 2018).

Embryo development and implantation is a key stage in early pregnancy, and involves the chronological process of maturation, fertilization, cleavage, and blastocyst formation, along with synchronized cell proliferation and differentiation. Concomitantly, an immunotolerant state is maintained at the maternal-fetal interface by human leukocyte antigen-G (HLA-G). Women with lower HLA-G expression have a higher risk of recurrent spontaneous abortion (RSA) and preeclampsia (PE) (Cecati et al., 2011), which can be attributed to increased infiltration of maternal immune cells. The placenta at the maternal-fetal interface is formed by cytotrophoblast cells (CTB), which invade the uterine tissue and migrate to the uterine spiral artery, wherein they further differentiate into endovascular trophoblast cells (Sultana et al., 2018). Trophoblast invasion and migration mediate immunotolerance and remodeling of the uterine spiral artery at the maternal-fetal interface during embryo implantation (Ren et al., 2020). Dysfunctional trophoblast phenotypes can lead to adverse pregnancy outcomes such as SA. In addition to HLA-G, matrix metalloproteinases (MMPs) and vascular endothelial growth factor (VEGF) are also dysregulated during SA. However, the post-transcriptional regulation of the above genes in the context of trophoblast function and SA have not been studied in detail.

N6-methyladenosine (m^6^A) is the most abundant RNA modification (Csepany et al., 1990) that stabilizes mRNAs and also modulates their localization, transport, and post-translational regulation (Lence et al., 2019). The methylation of adenosine is mediated by the methyltransferase(also known as “writers”) complex consisting of METTL3, METTL14, WTAP, KIAA1429, and RBM15/RBM15B. The m^6^A modification can be reversed by demethylases(also known as “erasers”) such as Fat mass and Obesity-associated protein (FTO) and AlkB homologue 5 (ALKBH5). The m^6^A can influence post-transcriptional gene expression during transcription through specific recognition by m6-binding proteins (also known as “readers”) (Huang et al., 2020). Currently, the study of FTO and its expression products has attracted extensive interest from scientists due to the identification of FTO as the first m^6^A-demethylase modified by m^6^A (Jia et al., 2011). Whole genome association studies have shown that the FTO gene is closely related to diabetes and obesity (Gerken et al., 2007). In addition, there is increasing evidence that the m^6^A modification plays an important role in various pathological conditions such as nervous system diseases, metabolic diseases, reproductive dysfunction diseases and cancer (Zhen et al., 2019). Li et al. (2019) recently showed that AlKbH5 affects the stability of *CYR61* mRNA in trophoblasts and is involved in the pathogenesis of SA. In addition, Andraweera et al. (2015) identified the FTO s9939609 single nucleotide polymorphism as a risk factor of SA in a cohort study of 202 Sinhalese women with a history of SA and 202 normal control women. Given the demethylase activity of FTO and aberrant trophoblast function in SA, we hypothesized that FTO is dysregulated in the trophoblasts during SA and results in the abnormal accumulation of transcripts with m^6^A. Our findings indicate that diminished FTO-mediated demethylation in trophoblasts is a likely pathological basis of SA.

## Materials and Methods

### Subjects

Eighty-four healthy women of average age 33 years and regular menstruation, including 49 with spontaneously aborted singleton pregnancies (SA) and 35 with voluntarily terminated pregnancies (normal), were recruited for the study. The SA group had received uterine curettage, and B-ultrasound at 8 weeks showed no fetal heart rate and fetal size inconsistent with the gestation time, which was indicative of spontaneous embryo abortion. In the normal group, the pregnancy sac and primitive heart tube were seen during the B-ultrasonic examination. There was no significant difference in age and number of pregnancies between the two groups. Chorionic villi samples (50–100 g) were collected from the SA group during the uterine curettage or at 6–9 weeks from the normal group, rinsed repeatedly with normal saline, and stored in liquid nitrogen. In addition, 5 mL blood samples were also collected from all subjects. This study was approved by the ethics committee of the First Affiliated Hospital of Guangzhou University of Traditional Chinese Medicine. All the patients signed the informed consent form.

### Immunofluorescence Assay

The villi tissue was frozen in liquid nitrogen and then sliced using a cryotome. The cryosections were incubated with rabbit anti-FTO (1:500, ab126605, Abcam), mouse anti-HLA-G (1:500, ab7759, Abcam) and mouse anti-m^6^A (1:500, ab208577, Abcam) antibodies, washed thrice with PBS, and then probed with the secondary antibody for 1h. After washing thrice with PBS, the sections were sealed with the coverslips and gelvatol, and observed under a fluorescence microscope (Leica TCS-SP5).

### Real-Time Quantitative Polymerase Chain Reaction (qRT-PCR)

The total RNA was extracted from frozen villi samples using Trizol reagent, and its purity was evaluated using a Nanophotometer (Implen, Germany). Following reverse transcription with the Thermo RevertAid First Strand cDNA Synthesis Kit (Thermo Fisher Scientific, United States), 10μl cDNA was amplified using Thermo Maxima SYBR Green qPCR Master Mix (Thermo Fisher Scientific, United States) on the CFX96 cycler (Bio-rad, United States). The RT-PCR parameters were as follows: 95°C for 10 min, followed by 40 cycles of 95°C for 10 s, 60°C for 34 s, and 95°C for 15 s. All primers were designed and synthesized by Sangon Bioengineering Company (Table 1). The expression levels of the target genes were calculated by the (2^–ΔΔ*CT*^) method.

**TABLE 1:** mRNA PCR primer.

Primer	Sequences (5′-3′)
FTO	Forward: GCCGCTGCTTGTGAGACCTTC
	Reverse: TGCTGCTCTGCTCTTAATGTCCAC
GAPDH	Forward: GCACCGTCAAGGCTGAGAAC
	Reverse: TGGTGAAGACGCCAGTGGA
HLA-G	Forward: AGAGGAGACACGGAACACCAAGG
	Reverse: CAGGTCGCAGCCAATCATCCAC
MMP2	Forward: GCCTCTCCTGACATTGACCTTGG
	Reverse: CACCACGGATCTGAGCGATGC
MMP7	Forward: CATGATTGGCTTTGCGCGAG
	Reverse: GCATCTCCTCCGAGACCTGT
MMP9	Forward: TCCTGGTGCTCCTGGTGCTG
	Reverse: CTGCCTGTCGGTGAGATTGGTTC
VEGFA	Forward: GCCTTGCCTTGCTGCTCTACC
	Reverse: GGTCTCGATTGGATGGCAGTAGC
VEGFR	Forward: CGGACAGTGGTATGGTTCTTGCC
	Reverse: GTGGTGTCTGTGTCATCGGAGTG
ALKBH5	Forward: CGGCGAAGGCTACACTTACG
	Reverse: CCACCAGCTTTTGGATCACCA
WTAP	Forward: CTTCCCAAGAAGGTTGATTGA
	Reverse: TCAGACTCTCTTAGGCCAGTTAC
YTHDF2	Forward: CCTTAGGTGGAGCCATGATTG
	Reverse: TCTGTGCTACCCAACTTCAGT
METTL3	Forward: TCTGGGGGTATGAACGGGTA
	Reverse: CTGGTTGAAGCCTTGGGGAT
IGFBP1	Forward: GGCGTCTCATTGGCAAGGAAGG
	Reverse: CTCAGGGTTGTAAAGGGTAAGGTCTTG
IGFBP2	Forward: CATCATCGGAAAGGAGGGCTTGAC
	Reverse: GCATGGATGGTGACAGGCTTCTC
IGFBP3	Forward: GCAAAGGATTCGGAAACTTCAGATACG
	Reverse: TCACAGCTCTCCACCACTCCATAC

### Enzyme Linked Immunosorbent Assay (ELISA)

The villi samples were homogenized in PBS and centrifuged at 2000 *g* for 10min at 4°C. The supernatants were collected, and the levels of MDA, SOD, and FTO were detected using specific ELISA kits (MDA, SOD from R&D, United States, and FTO kit from Biovision, United States).

### Western Blotting

The villi tissues were lysed, and the amount of protein in the lysates was determined using the BCA Kit (Beyotime Biotechnology, China). Equal amounts of protein per sample were denatured in the loading buffer at 100°C for 5 min. The samples were electrophoresed in SDS-PA gel, and transferred to NC membrane. After blocking with 5% skimmed milk at room temperature for 1 h, the blots were incubated overnight with anti-FTO monoclonal antibody (ab126605) at 4°C, washed thrice with TBST, and incubated with secondary antibody for 1h at room temperature. The following antibodies were used for WB:METTL3 (ab195352), WTAP (ab195380), YTHDF2 (ab220163), IGF2BP1 (ab184305), and IGF2BP2 (ab124930). The positive bands were developed using the enhanced luminescent reagent (Bio-Rad, United States) and observed on an imaging system (Bio-Rad, United States).

### RNA Immunoprecipitation

RNA Immunoprecipitation was performed using the EZ-Magna RIP^TM^ RNA-Binding Protein Immunoprecipitation Kit (Merck, Germany) according to the manufacturer’s protocol. Briefly, the villi tissues were washed thrice with ice-cold PBS and centrifuged. The pelleted cells were resuspended in 200 μl RIP buffer and incubated with magnetic antibody-conjugated A/G beads at 4°C for 3 h. One hundred microliter of the supernatant was diluted with 900 μl RIP buffer and incubated with proteinase K. The FTO-bound RNA was then extracted using the phenol/chloroform method for RIP-Seq, and the levels of *HLA-G*, *MMP2*, *MMP7*, *MMP9*, *VEGFA*, and *VEGFR* mRNAs were quantified by qRT-PCR. The following antibodies were used for RIP and MeRIP: FTO (ab126605), m^6^A (ab208577), YTHDF2(ab220163), and rabbit anti-human IgG (1: 100, 10285-1-AP). The cells were incubated with the antibodies at room temperature for 30 min. The primers for RT-qPCR are listed in Table 1.

### RNA m^6^A Dotblot Assays

Firstly, the total RNA of chorionic villi was extracted with trizol reagen and treated with DNase I to remove possible DNA contamination. The ratio of Poly (A) + RNA was diluted to 2 and 60 ng and spotted onto the nylon film (GE Healthcare, China). Then UV-crosslinked, blocking buffer was used to seal. The cells were incubated with m^6^A antibody (ab232905) overnight, then incubated with horseradish peroxidase labeled Goat Anti-Rabbit IgG H&L (ab6721) for 1 h, and finally detected by DAB peroxidase substrate kit (Yeasen Biotechnology, China).

### Statistical Analysis

SPSS19.0 and GraphPad Prism6.0 were used for all statistical analysis. The measurement data were expressed as mean ± standard deviation ($\bar{x}$ ± s) of three independent experiments. One-way analysis of variance and LSD were used to compare groups with normally distributed data. Non-parametric test (Kruskal-Wallis test) was used for data with non-normal distribution. *P* < 0.05 was considered statistically significant.

## Results

### Placental Oxidative Stress in SA Is Associated With Aberrant FTO Expression in the Chorionic Villi and Trophoblasts

**FIGURE 1:**
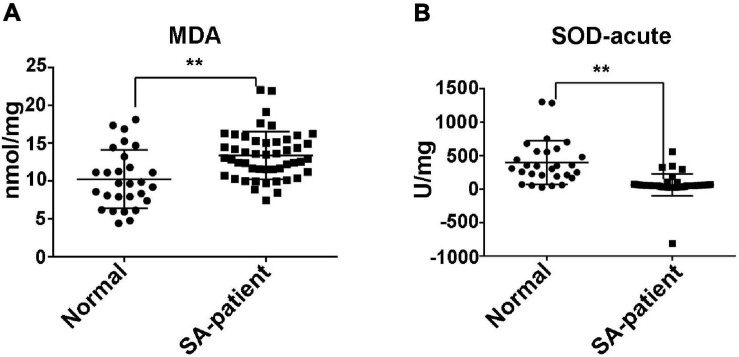
**(A)** The expression of MDA in the villi of N and SA groups. **(B)** The expression of SOD in the villi of N and SA groups (N, normal, *n* = 28; SA, spontaneous abortion, *n* = 48), **P < 0.01.

**FIGURE 2:**
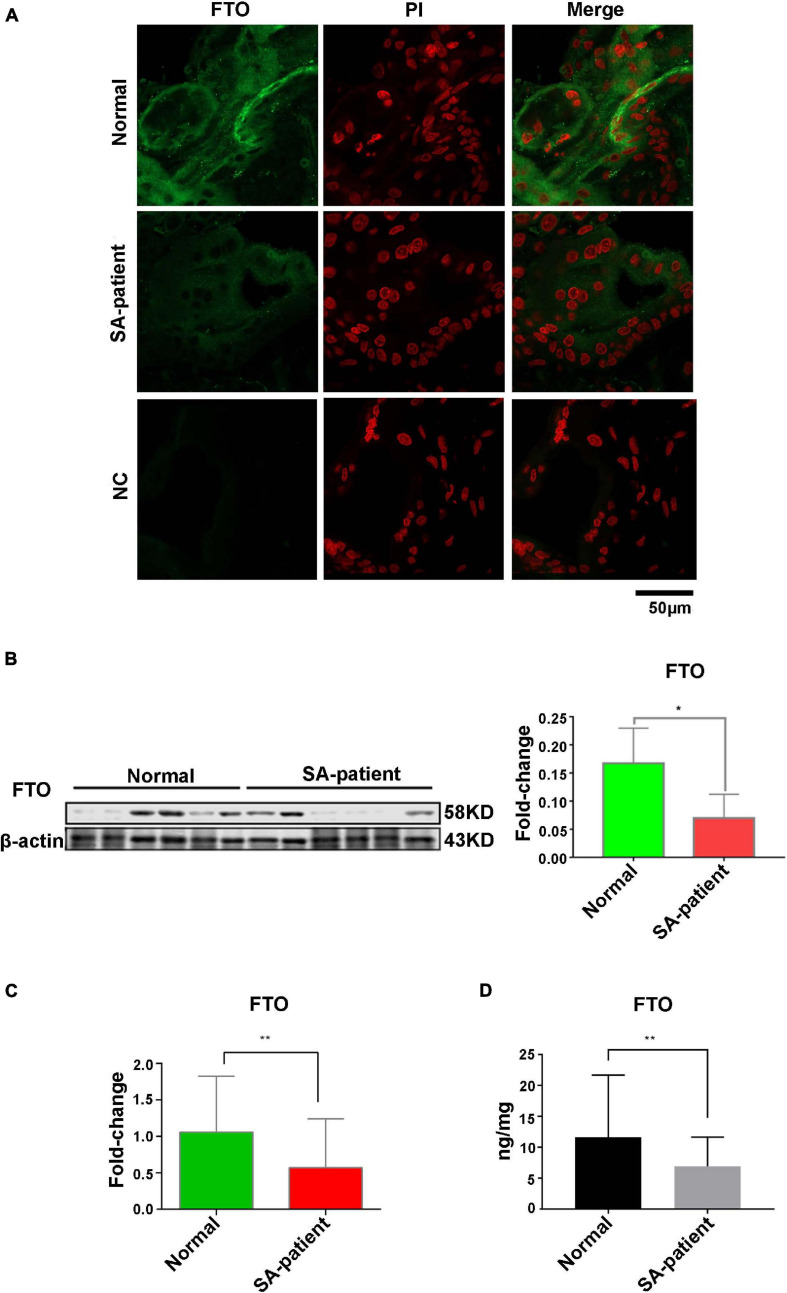
**(A)** Representative immunofluorescence images of chorionic villi showing in situ expression of FTO in both groups. Green, FTO; Red, PI counterstained nuclei. Normal *n* = 3; Patient *n* = 3; NC, negative control *n* = 3. **(B)** Immunoblot showing expression level of FTO protein in the villi of two groups (Normal *n* = 6; Patient *n* = 6), *P<0.05. **(C)** The expression level of *FTO* mRNA in villi of two groups (Normal *n* = 30; Patient *n* = 67), **P<0.01. **(D)** The expression level of FTO in the villi of two groups as detected by ELISA (Normal *n* = 28; Patient *n* = 48), **P<0.01.

Compared to normal pregnancies, MDA levels were significantly higher in the villi of the SA group (*P* < 0.05) and the SOD level was markedly decreased (Figure 1). Furthermore, The *FTO* mRNA and protein levels were significantly decreased in the chorionic villi of the SA group compared to the controls (Figures 2A–C). Moreover, ELISA was performed to detect the FTO expression in larger samples of patients in villi of two groups, showing the FTO expression was significantly downregulated in SA patients (Figure 2D).

### Abnormal Gene Expression Levels of RNA m^6^A Modification-Related Proteins in SA Patients

**FIGURE 3:**
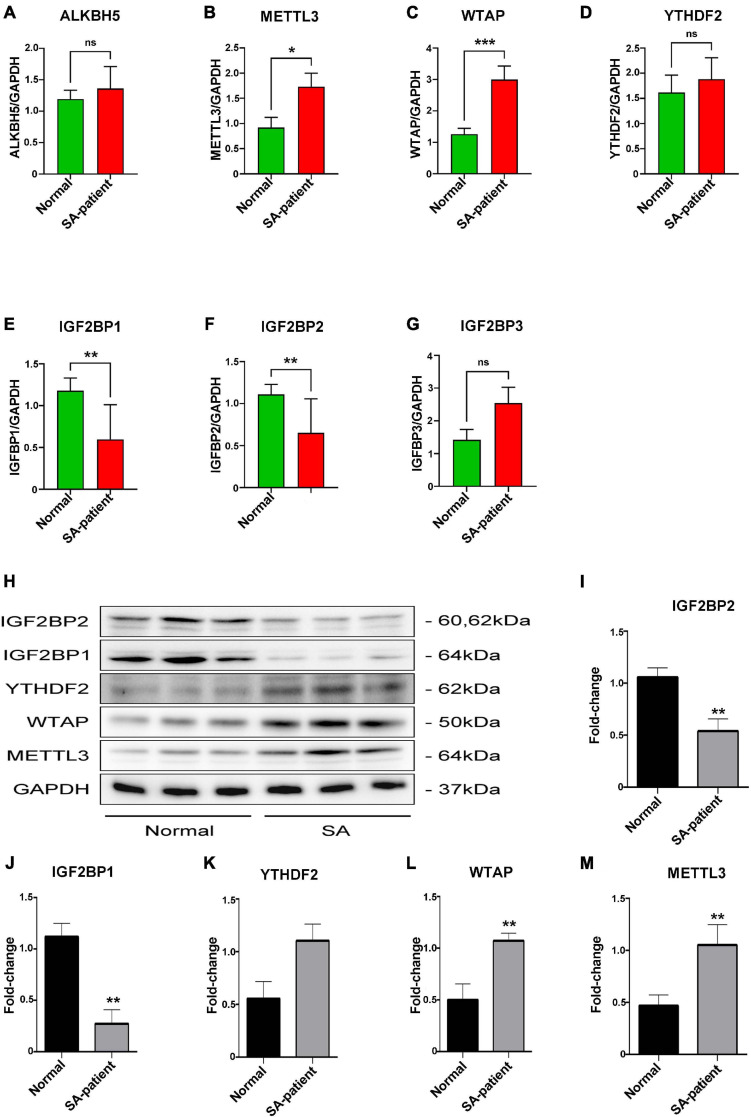
**(A)** *ALKBH5*, **(B)** *METTL3*, **(C)** *WTAP*, **(D)** *YTHDF2*, **(E)** *IGF2BP1*, **(F)** *IGF2BP2*, and **(G)** *IGF2BP3* mRNA levels in the indicated groups (Normal *n* = 8; Patient *n* = 8, *P<0.05, **P<0.01). **(H)** Immunoblot showing expression level of target protein in the villi of two groups. **(I)** IGF2BP2, **(J)** IGF2BP1, **(K)** YTHDF2, **(L)** WTAP, and **(M)** METTL3 protein expression level in the villi of two groups (Normal *n* = 6; Patient *n* = 6). *P<0.05, **P<0.01, ***P < 0.001.

To explore whether mRNA m^6^A methylation is involved in the pathogenesis of SA, we screened samples with differential FTO expression according to the FTO levels at enrolment and analyzed the expression of m^6^A “writers,” “erasers,” and “readers” in the villi tissues in the above SA patients (*n* = 8) and normal (*n* = 8) using qRT-PCR and Western blot (Figures 3H). Compared to the normal patients, the expression levels of *METTL3* and *WTAP* were significantly elevated (*p* < 0.05, Figures 3B,C,L,M) while the expression levels of *IGF2BP1*, *IGF2BP2* were significantly decreased (*p* < 0.05, Figures 3E,F,I,J) in SA patients. However, *ALKBH5*, *YTHDF2*, and *IGF2BP3* were elevated with no statistical significance between groups (Figures 3A,D,G,K).

### Upregulation of m^6^A Modification in the Villi of SA Patients

**FIGURE 4:**
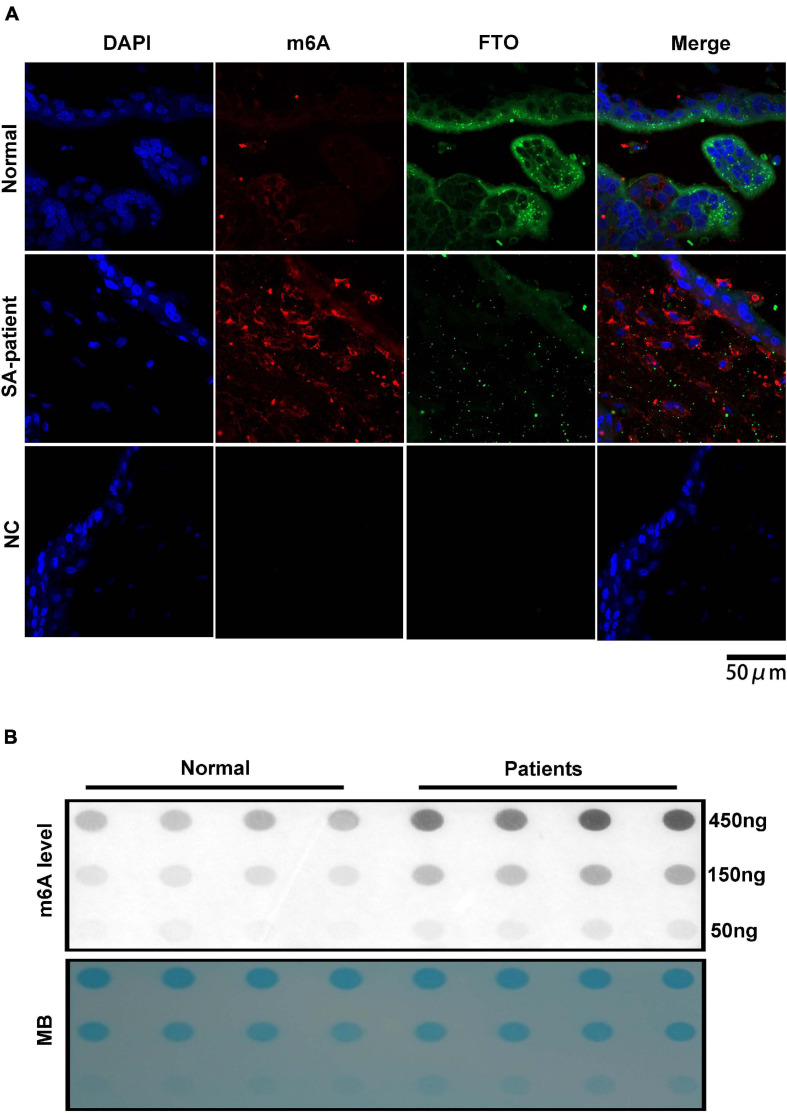
**(A)** Representative immunofluorescence images of chorionic villi showing in situ expression of FTO and m^6^A in both groups (Normal *n* = 3; Patient *n* = 3; NC:negative control *n* = 3). Blue – FTO and red – m^6^A. **(B)** Poly(A)+ RNAs isolated from chorionic villi were used in dot blot analyses with m^6^A antibody. RNA was loaded by equal dilution method (Normal *n* = 4; Patient *n* = 4).

Fat mass and obesity can catalyse and mediate the demethylation of m^6^A, therefore, downregulated FTO in the villi of SA patients may lead to the disorder of m^6^A modification. Double-immunofluorescence staining (FTO and m^6^A) was performed for each clinical sample and revealed that the FTO expression in the villi of SA patients was downregulated, while the m^6^A expression was upregulated, with a negative correlation between them (Figure 4A). Then, we extracted the RNA of chorionic villi from each sample of SA patients and normal pregnancy control group and performed RNA m^6^A Dotblot test after multiple dilution. We found that the content of m^6^A in Patients group was significantly up-regulated while keeping the same amount of samples (Figure 4B).

### Phenotypic Differences of Villi Between SA Patients and Normal Pregnant Women

**FIGURE 5:**
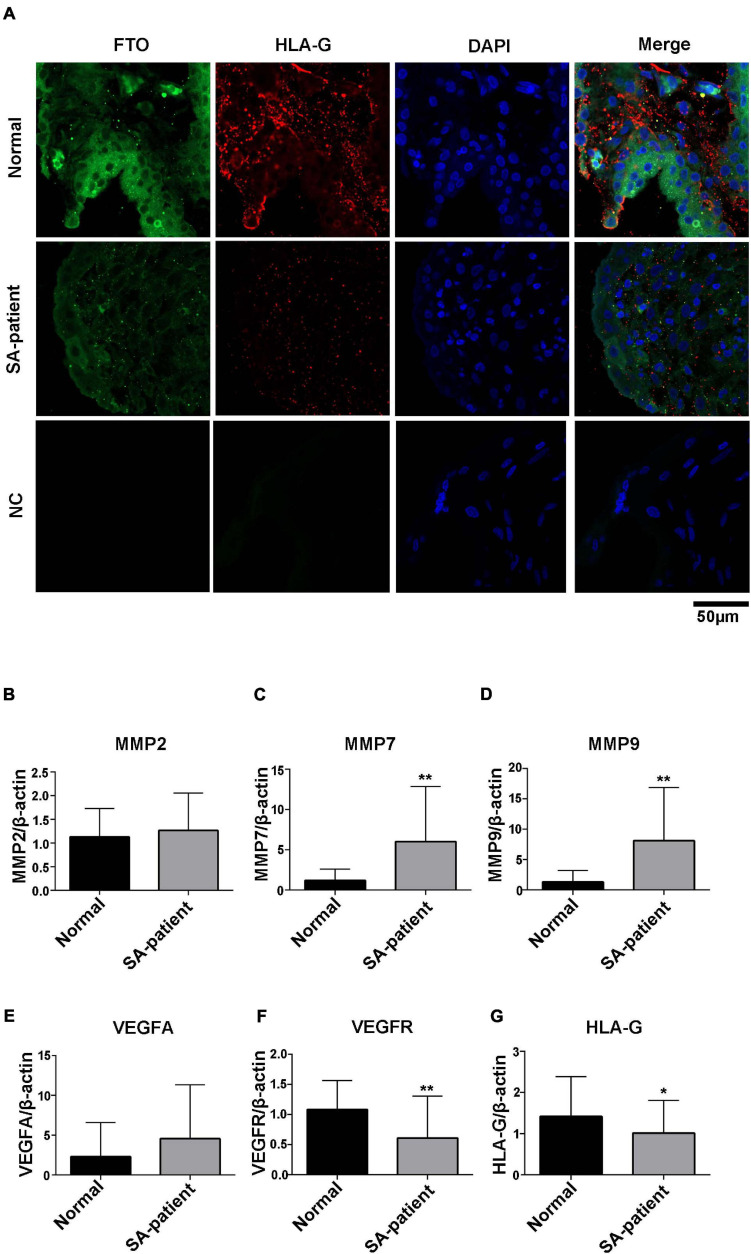
**(A)** Representative immunofluorescence images of chorionic villi showing in situ expression of FTO and HLA-G in both groups (Normal *n* = 3; Patient *n* = 3; NC:negative control *n* = 3), green – FTO and red – HLA-G. **(B)** *MMP2*, **(C)** *MMP7*, **(D)** *MMP9*, **(E)** *VEGFA*, **(F)** *VEGFR*, and **(G)** *HLA-G* mRNA levels in the indicated groups. (Normal *n* = 35; Patient *n* = 49, *P<0.05 and **P<0.01).

As shown in Figure 5A, the in situ expression of FTO and HLA-G was significantly lower in the trophoblasts of the SA patients, and the expression pattern of both were similar. Finally, *HLA-G* and *VEGFR* mRNA levels were downregulated in the SA group (*p* < 0.05, Figures 5G,F), and *MMP7* and *MMP9* were upregulated (*p* < 0.05, Figures 5C,D). *MMP2* and *VEGFA* mRNA levels were also higher in the SA group, although the difference was not statistically significant (Figures 5B,E).

### FTO Downregulation Increases the m^6^A Modification of Several Genes During SA

**FIGURE 6:**
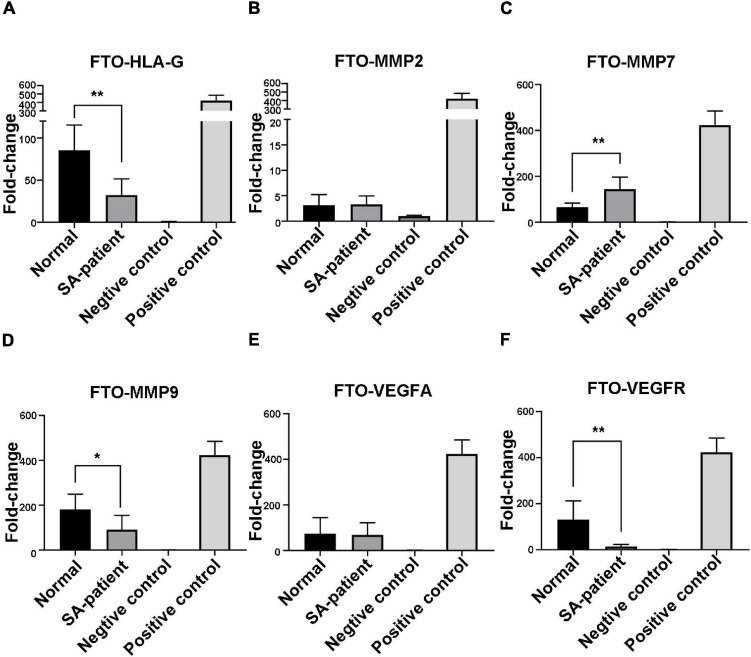
Fold-change in FTO-bound **(A)** *HLA-G*, **(B)** *MMP2*, **(C)** *MMP7*, **(D)** *MMP9*, **(E)** *VEGFA*, and **(F)** *VEGFR* mRNA in the chorionic villi of the indicated groups (Normal *n* = 9; Patient *n* = 9, *P < 0.05, **P < 0.01).

**FIGURE 7:**
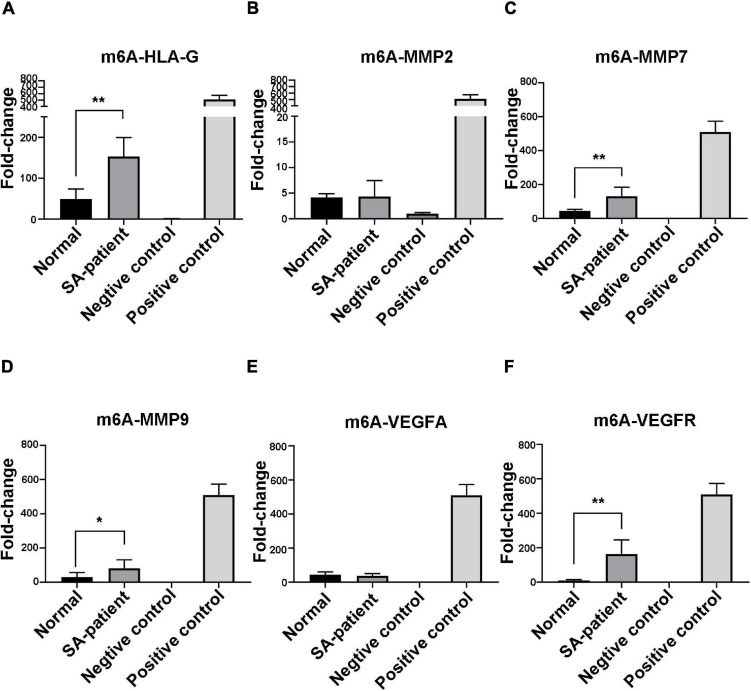
Fold-change in m^6^A-bound **(A)** *HLA-G*, **(B)** *MMP2*, **(C)** *MMP7*, **(D)** *MMP9*, **(E)** *VEGFA*, and **(F)** *VEGFR* mRNA in the chorionic villi of the indicated groups (Normal *n* = 9; Patient *n* = 9, *P < 0.05, **P < 0.01).

**FIGURE 8:**
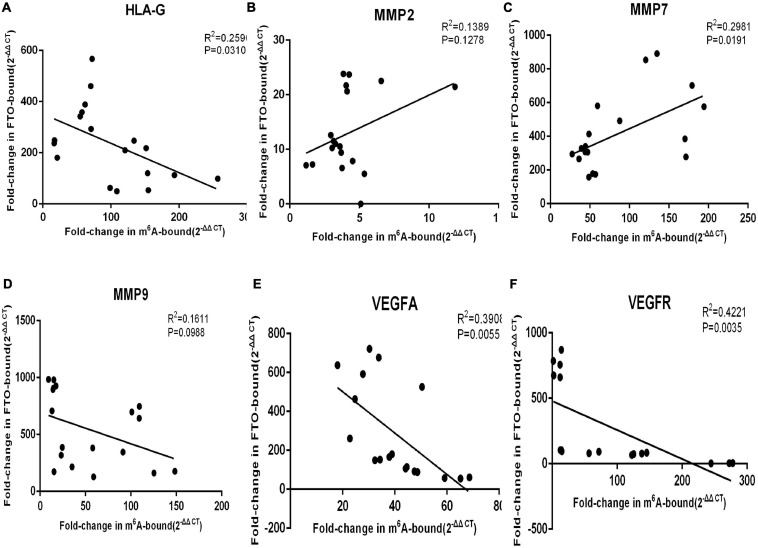
The levels of FTO-bound **(A)** *HLA-G*, **(B)** *MMP2*, **(C)** *MMP7*, **(D)** *MMP9*, **(E)** *VEGFA*, and **(F)** *VEGFR* mRNA was correlated to these mRNA with m^6^A modification.

**FIGURE 9:**
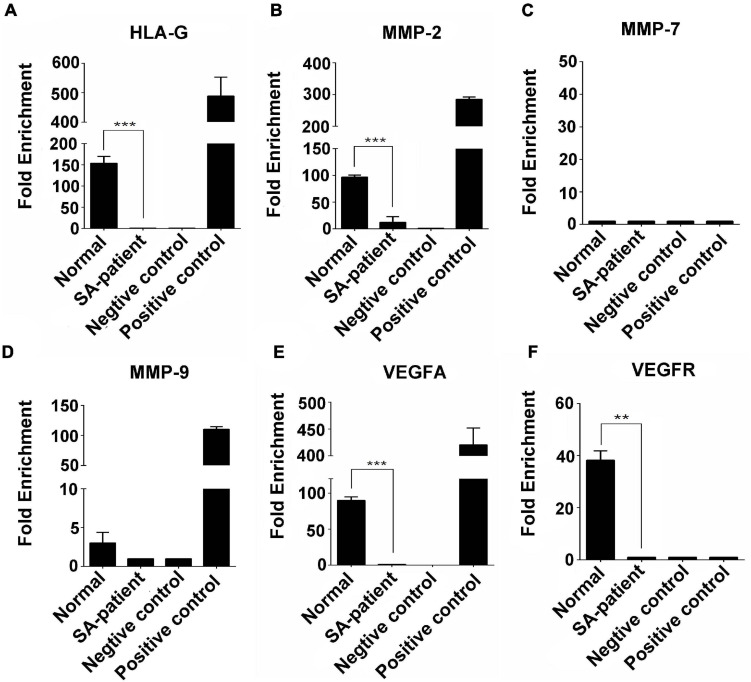
Fold-change in YTHDF2-bound **(A)** *HLA-G*, **(B)** *MMP2*, **(C)** *MMP7*, **(D)** *MMP9*, **(E)** *VEGFA*, and **(F)** *VEGFR* mRNA in the chorionic villi of the indicated groups. (Normal *n* = 9; Patient *n* = 9, **P < 0.01, ***P < 0.001).

Immunoprecipitation and qPCR experiments showed a significant decrease in the levels of FTO-bound *HLA-G*, *VEGFR* and *MMP9* mRNA (Figures 6A,D,F), and markedly higher enrichment of FTO-bound *MMP7* (Figure 6C) in the SA patients compared to the normal pregnant women. No significant difference was observed in the levels of co-precipitated *MMP2* and *VEGFA* between the two groups (Figures 6B,E). Consistent with this, the SA samples showed have a higher enrichment ratio of *HLA-G*, *VEGFR*, *MMP7* and *MMP9* mRNA with m^6^A modification (Figures 7A,C,D,F), while that of the modified *MMP2* and *VEGFA* mRNAs were unaffected (Figures 7B,E). Linear correlation analysis showed that the levels of FTO-bound *HLA-G*, *MMP2*,*MMP7*,*MMP9*,*VEGFA* and *VEGFR* mRNA was correlated with these mRNA with m^6^A modification (Figures 8A–F). Immunoprecipitation and qPCR experiments both showed that the target protein YTHDF2 directly bound to *HLA-G*, *VEGFA*, *VEGFR*, *MMP9*, and *MMP2* mRNA, but almost unbound to *MMP7* mRNA (Figure 9C). Compared with normal pregnant women, the levels of *HLA-G*, *VEGFA*, *VEGFR*, and *MMP2* mRNA bound by YTHDF2 were significantly decreased in SA patients (Figures 9A,B,E,F). However, the level of co-precipitated *MMP9* was not significantly different between groups (Figure 9D).

Taken together, SA is associated with increased methylation of genes involved in immunotolerance, immune cell infiltration and angiogenesis at the maternal-fetal interface.

## Discussion

We found that the RNA demethylase FTO was downregulated in the chorionic villi of women that underwent SA, and correlated with oxidative stress and aberrant m^6^A accumulation at the maternal-fetal interface. During the implantation of embryos, genetic material is transferred through the dynamic regulation of methylation and demethylation (Qin et al., 2020). Typically, RNA methylation regulates gene expression after transcription. Interestingly, we found that the m^6^A-modified writer proteins, including METTL3 and WTAP, were elevated when the demethylase FTO was downregulated in SA patients. Meanwhile, another eraser protein (ALKBH5) did not affect the abnormal accumulation of m^6^A. Therefore, we suggest that the aberrant accumulation of m^6^A in SA was also related with the dysregulation of other modifiers. It is generally believed that when m^6^A RNA is methylated, demethylase FTO will play a role after methylated transferase (Mathiyalagan et al., 2019). Recent studies have correlated changes in m^6^A levels with human reproductive disorders (Kaspi et al., 2018). Given that the m^6^A modification occurs at the 3’-UTR near the transcription termination site (Lan et al., 2019), accumulation of hypermethylated mRNAs due to aberrant FTO expression during SA likely affects the expression levels of crucial genes.

Oxidative stress in the placenta and synthetic trophoblast cells can induce abortion (Fortis et al., 2018). We detected lower levels of the antioxidant enzyme SOD in the homologous villi tissue of the SA group compared to the controls, which is consistent with the findings of Popovia et al. (El-Far et al., 2007). In agreement with Hempstock et al. (El-Far et al., 2009; Zhao et al., 2020), MDA levels were significantly higher in the chorionic villi of the SA group. MDA is a by-product of lipid peroxidation, and its elevated content reflects excessive production of lipid peroxides or impaired antioxidant defence mechanisms. A previous study showed that aberrant m^6^A modification disrupted the antioxidant system in preadolescent testicular injury (Hernández-Vargas et al., 2020). Likewise, the oxidative stress during early and late SA (Costello et al., 2019) has been correlated with decreased expression of HLA-G and VEGF along with elevated MMPs in the chorionic villi, which can impair immunotolerance, trophoblastic invasion and angiogenesis at the maternal-fetal interface. Consistent with previous reports, *HLA-G* and *VEGFR* were significantly downregulated in the SA group, while *MMP7* and *9* were upregulated.

To further elucidate whether impaired demethylation due to FTO inhibition contributes to the pathogenesis of SA, we analyzed the levels of FTO- and m^6^A-bound *HLA-G, VEGFR* and *MMP9* mRNA in the trophoblasts. As expected, the mRNAs showed decreased binding to FTO, which was accompanied by increased methylation as indicated by anti-m^6^A antibody-mediated enrichment of the specific transcripts. Since m^6^A accumulation in the 5’UTR region of mRNA inhibits translation (Zhou et al., 2018; Sun et al., 2020), the downregulation of these genes observed in SA samples can be attributed to their hypermethylated state.

As mentioned before, m^6^A from SA is abnormally deposited on RNA transcripts during transcription due to dysregulation of writer and eraser, and therefore affects gene expression post-transcriptionally by altering the specific recognition of m^6^A binding proteins (also called readers) (Shi et al., 2019). The IGF2BP family protects m^6^A-modified mRNA from degradation and promotes mRNA translation (Huang et al., 2018), whereas we found that compared to normal individuals, *IGF2BP1/2* was less expressed using qPCR and m^6^A itself is abnormally accumulated in SA patients. Thus, it is hard to tell that the abnormalities of the phenotypic genes in SA patients were due to the reduced IGF2BPs or hypermethylation of m^6^A. Accordingly, we did not select IGF2BPs for RIP-qPCR. On the other hand, YTHDF2 functions to bring m^6^A-modified translatable mRNAs to the decay site of mRNAs and mediates the decay of mRNAs (Lee et al., 2020). Our analysis revealed reduced binding of YTHDF2 to *HLA-G*, *VEGFR*, and *MMP9* in SA patients, accompanied by increased m^6^A methylation. Besides, qPCR found no difference of YTHDF2 expression between normal and SA patients, suggesting that the downregulation of these genes in SA patients is due to their hypermethylation status.

Interestingly, the levels of both FTO- and m^6^A-bound *MMP7* were significantly increased in the SA group, which indicates that *MMP7* methylation is regulated by other mechanisms. At the same time, we found elevated IGF2BP1-2 in both SA groups, suggesting that the abnormal accumulation of methylation in MMP7 is largely responsible for the abnormal elevated recognition of writer proteins. Whether the level of FTO and m^6^A RNA methylation in RNA transcripts affect the recognition and functions of different m^6^A reader proteins remains elusive (Shi et al., 2019). In addition, the changes in *MMP2* and *VEGFA* methylation levels were not significantly affected in the SA samples, which also points to other regulatory mechanisms. Therefore, it is necessary to identify the core pathological genes of SA, and to explore the function of each m^6^A-regulated gene to explore the potential molecular mechanisms. Nevertheless, our findings show that impaired mRNA demethylation at the maternal-fetal interface due to low FTO levels can at least partly mediate SA pathogenesis. A dysfunctional placenta can significantly increase the risk of preeclampsia, fetal growth restriction (FGR) and early SA (Rana et al., 2020; Yang et al., 2021). Based on our results, we recommend analysis of placental FTO demethylase levels in patients with late SA in order to determine whether these epigenetic changes are sudden or related to implantation. Beside, FTO downregulation increases the m^6^A modification tends to be incomplete, and further studies of the mechanism of FTO and m^6^A methylation in vitro experiments is recommended.

## Conclusion

The villi of SA patients are impaired in immune tolerance, invasion and migration, and angiogenesis, manifested as decreased *HLA-G* and *VEGFR* mRNA expression and increased *MMP7* and *MMP9* mRNA expression. The villi of SA patients were in a state of oxidative stress, with downregulated FTO. Meanwhile, the combination of relevant phenotypic functional genes was downregulated but the m^6^A modification was increased. In conclusion, inhibition of the RNA demethylase FTO leads to hypermethylation of crucial genes and oxidative stress in the maternal-fetal interface during SA.

## Data Availability Statement

The original contributions presented in the study are included in the article/supplementary material, further inquiries can be directed to the corresponding authors.

## Ethics Statement

The studies involving human participants were reviewed and approved by the Ethics Committee of the First Affiliated Hospital of Guangzhou University of Traditional Chinese. The patients/participants provided their written informed consent to participate in this study.

## Author Contributions

WQ, SL, and JG conceived and designed the experiments, and wrote the manuscript. WQ, YZ, HW, XL, ZR, and LY performed the experiments. QY, LZ, HT, and JL analyzed the data. All authors contributed to the article and approved the submitted version.

## Conflict of Interest

The authors declare that the research was conducted in the absence of any commercial or financial relationships that could be construed as a potential conflict of interest.
